# Association between dietary index for gut microbiota and self-reported severe headache or migraine in U.S. adults: a cross-sectional study from NHANES

**DOI:** 10.3389/fnut.2025.1549251

**Published:** 2025-03-31

**Authors:** Jingjing Liu, Hailian Liu, Weiyi Li, Shaoqiang Huang

**Affiliations:** Department of Anesthesiology, Obstetrics and Gynecology Hospital of Fudan University, Shanghai, China

**Keywords:** cross-sectional study, dietary index for gut microbiota, DI-GM, headache, migraine, NHANES

## Abstract

**Background:**

The diet–microbiota–gut–brain axis is an emerging frontier in brain health, with microbiota-targeted dietary interventions offering potential benefits. This study aimed to explore the association between the recently introduced dietary index for gut microbiota (DI-GM) and self-reported severe headache or migraine in U.S. adults.

**Methods:**

This cross-sectional study analyzed the National Health and Nutrition Examination Survey (NHANES) data from 1999 to 2004. Severe headache or migraine was determined based on participants’ responses to the question: “Have you had a severe headache or migraine in the past 3 months?” The DI-GM was calculated from dietary recall data. Multivariable logistic regression models were performed to evaluate the odds ratio (OR) and 95% confidence interval (95% CI) for the association between DI-GM and severe headache or migraine. Secondary analyses included restricted cubic splines (RCS) and subgroup analyses.

**Results:**

After adjustments, a higher DI-GM score and BGMS were associated with a lower prevalence of severe headache or migraine (DI-GM: OR = 0.95, 95% CI = 0.91–0.99, *p* = 0.011; BGMS: OR = 0.90, 95% CI = 0.85–0.96, *p* = 0.003). RCS showed a linear relationship between DI-GM and severe headache or migraine. In two-piecewise regression models, the adjusted OR for developing a severe headache or migraine was 0.90 (95% CI = 0.85–0.97, *p* = 0.005) in participants with a DI-GM score ≥ 4, whereas no association was observed in those with a DI-GM score < 4.

**Conclusion:**

The DI-GM was negatively associated with the prevalence of self-reported severe headache or migraine in U.S. adults, particularly when scores exceeded 4.

## Introduction

Migraine is a neurological disorder characterized by recurrent moderate to severe headaches lasting 4–72 h, often accompanied by nausea, vomiting, photophobia, phonophobia, and sometimes aura with reversible neurological symptoms like visual disturbances (1). According to the Global Burden of Diseases, Injuries, and Risk Factors Study (GBD) 2021, migraine and other headache disorders accounted for 5.2% of all years lived with disability (YLDs) globally, placing it as the third leading cause of YLDs (2). Despite advances in healthcare, migraine remains a significant contributor to non-fatal health loss, disproportionately impacting individuals during their most productive years and causing considerable socioeconomic burdens (3).

Previous studies have highlighted the central role of gut microbiota in migraine pathogenesis, mediating neuroinflammation (e.g., via the vagus nerve and cytokines), gut-brain signaling (e.g., through gamma-aminobutyric acid), and metabolic functions (e.g., via short-chain fatty acids [SCFAs]) (4–6). Moreover, diets modulate gut microbiota, as exemplified by fermented foods that steadily increase microbiota diversity and decrease inflammatory markers, and dietary fiber that promote SCFAs production (7–9). In addition, dietary approaches could serve as effective strategies for the prophylaxis of headache or migraine (10, 11). Therefore, microbiota-targeted dietary interventions present a promising approach for migraine management. The dietary index for gut microbiota (DI-GM), developed by Kase et al. (12) through a comprehensive review and validated via its association with biomarkers of gut microbiota diversity, assesses dietary impacts on gut microbiota using 14 specific foods or nutrients, with higher scores indicating healthier gut microbiota. The diet–microbiota–gut–brain axis is an emerging frontier in brain health, with microbiota-targeted dietary interventions increasing recognized as critical (13). Therefore, DI-GM provides a valuable criterion for studying the diet-gut microbiota interplay and health, enabling more precise dietary recommendations. However, studies exploring the relationship between DI-GM and migraine remain scarce.

Chronic migraine is frequently comorbid with anxiety and mood disorders, particularly depression, which are considered disorders of the gut-brain axis (14). Zhang et al. (15) found that DI-GM was negatively associated with the prevalence of depression and its specific symptoms, including sleep disturbances, fatigue, and appetite changes. Consequently, this cross-sectional study aimed to investigate the association between DI-GM and severe headache or migraine using adult data from the National Health and Nutrition Examination Survey (NHANES), paving the way for microbiota-targeted dietary interventions in managing severe headache or migraine.

## Methods

### Study population

NHANES, a cross-sectional survey database conducted by the National Center for Health Statistics (NCHS), assesses the health and nutritional status of the non-institutionalized US population using a complex, stratified, multistage probability sampling method to select a nationally representative sample. Methodological details and survey design for NHANES are available at https://wwwn.cdc.gov/nchs/nhanes/. Publicly available data from 3 consecutive NHANES cycles (1999–2004) were used for this study, as they provided information on both DI-GM and severe headache or migraine. The NHANES procedures and protocols were approved by the Research Ethics Review Committee of the NCHS, and written informed consent was obtained from all participants.

**Figure 1:**
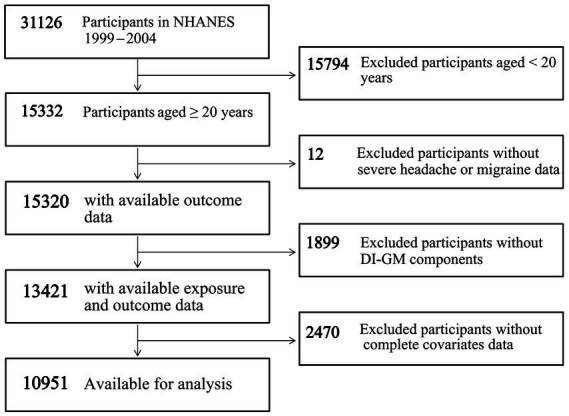
Flowchart of participant screening in NHANES 1999–2004. Abbreviations: DI-GM, dietary index for gut microbiota; NHANES, National Health and Nutrition Examination Survey.

Our study involved a total of 31,126 participants from 1999 to 2004. The exclusion criteria included participants aged < 20 years (*n* = 15,794), missing data on severe headache or migraine (*n* = 12), missing data on the DI-GM components (*n* = 1,899), and missing data on covariates (*n* = 2,470). A total of 10,951 participants were included in the final analysis, as shown in Figure 1.

### Headache assessment

Severe headache or migraine was determined based on participants’ self-reported responses to the question: “Have you had a severe headache or migraine in the past 3 months?” Respondents answering “Yes” were categorized as having severe headache or migraine, while those answering “No” were classified as not having it.

### DI-GM assessment

The DI-GM comprises 14 dietary components, including fermented dairy, chickpeas, soybean, whole grains, fiber, cranberries, avocados, broccoli, coffee, and green tea (unavailable due to NHANES not recording the specific type of tea consumption) as beneficial to gut microbiota, and refined grains, red meat, processed meat, and a high-fat diet (≥40% energy from fat) as unfavorable to gut microbiota (12). The DI-GM score was calculated utilizing 24-h dietary recall data derived from NHANES 1999–2004. For each participant, a score of 1 was assigned if the consumption of a beneficial component was above the sex-specific median, and 0 if below, with the sum forming beneficial to gut microbiota score (BGMS, ranges from 0 to 9); similarly, a score of 1 was assigned if the consumption of an unfavorable component was below the sex-specific median or if the high-fat diet component contributed less than 40% of total energy intake, and 0 if above or if dietary fat intake exceeded 40%, with the sum forming unfavorable to gut microbiota score (UGMS, ranges from 0 to 4). The DI-GM score was calculated by summing individual component scores, with a range of 0–13.

### Potential covariates

Several potential confounding variables were included based on published research findings and clinical relevance, including age, sex, race/ethnicity, marital status, education level, body mass index (BMI), physical activity, smoking status, alcohol consumption, comorbidities (diabetes and hypertension), and nutritional factors (vitamins B1, B2, B6, B12, zinc, copper) (16–24). Age was categorized as ≤45 years and >45 years. Race/ethnicity was classified into non-Hispanic White, non-Hispanic Black, Mexican American, and other races (16). Marital status was categorized as married or other (25). Education level was divided into 3 categories: less than high school, high school or equivalent, and above high school (15). Self-reported alcohol consumption was categorized as follows: never (<12 lifetime drinks), former (≥12 drinks in 1 year but no drinks in the last year or ≥12 lifetime drinks but no drinks in the past year), light (≤2 drinks per day for males and ≤1 drink per day for females), moderate (3 drinks per day for males and 2 drinks per day for females), and heavy (≥4 drinks per day for males and ≥3 drinks per day for females) (15). Smoking status was classified as yes (≥100 lifetime cigarettes) or no (<100 lifetime cigarettes) (26). Physical activity was categorized as yes (moderate or vigorous activity) or no (no moderate or vigorous activity) (27). Average blood pressure was calculated by the following protocol: the diastolic reading with zero is not used to calculate the diastolic average; if all diastolic readings were zero, then the average would be zero; if only one blood pressure reading was obtained, that reading is the average; if there is more than one blood pressure reading, the first reading is always excluded from the average (28). Hypertension was diagnosed when systolic ≥140 mmHg or diastolic ≥90 mmHg. The diagnostic criteria for diabetes include a diagnosis from a doctor, glycohemoglobin (HbA1c) levels >6.5%, fasting glucose levels ≥7.0 mmol/L, random/2-h oral glucose tolerance test (OGTT) blood glucose levels ≥11.1 mmol/L, or use of diabetes medication/insulin (28).

### Statistical analysis

Continuous variables were expressed as means ± standard deviation (SD), and differences between groups were assessed using one-way analysis of variance (ANOVA). Categorical variables were presented as numbers (percentages [%]), with group comparisons performed using the chi-square test.

The association between DI-GM and severe headache or migraine was evaluated using multivariable logistic regression models, providing odds ratios (ORs) and 95% confidence intervals (CIs). Model 1 was unadjusted, while Model 2 adjusted for age, sex, race/ethnicity, education level, marital status, and BMI. Model 3 further adjusted for physical activity, smoking status, alcohol consumption, diabetes, and hypertension. In Model 4, additional adjustments were made for vitamins B1, B2, B6, B12, zinc, and copper. Restricted cubic spline (RCS) were employed to explore potential non-linear dose–response relationships between DI-GM and severe headache or migraine. A two-piecewise logistic regression model was developed to assess the relationship between DI-GM and severe headache or migraine. Furthermore, to examine the robustness of the association, subgroup analyses were performed based on age (≤45 years versus >45 years), sex (male versus female), race/ethnicity (non-Hispanic White versus other), education level (less than college versus college or above), marital status (married versus other), smoking status (no versus yes), alcohol consumption (never/former versus current), physical activity (no versus yes), diabetes (no versus yes), hypertension (no versus yes).

All statistical analyses were conducted using R software (version 4.3.3). A two-sided *P*-value < 0.05 was considered statistically significant.

## Results

### Characteristics of the included participants

Of the 10,951 participants, 2,232 (20.4%) reported severe headache or migraine, while 8,719 (79.6%) did not. As shown in Table 1, participants with severe headache or migraine were more likely to be younger, female, non-White, less educated, unmarried, higher BMI, engage in less physical activity, be never drinkers, and have lower DI-GM score, BGMS and intake of vitamins B1, B2, B6, B12, zinc, and copper.

**Table 1:** Characteristics of participants without or with headache in NHANES 1999–2004.

Characteristic	Overall	Without headache	Headache	*P*-value
Participants, *n* (%)	10,951	8,719 (79.6)	2,232 (20.4)	
Age, *n* (%)				<0.001
≤45 years	5,053 (46.1)	3,719 (42.7)	1,334 (59.8)	
>45 years	5,898 (53.9)	5,000 (57.3)	898 (40.2)	
Sex, *n* (%)				<0.001
Male	5,245 (47.9)	4,504 (51.7)	741 (33.2)	
Female	5,706 (52.1)	4,215 (48.3)	1,491 (66.8)	
Race/ethnicity, *n* (%)				<0.001
Non-Hispanic White	5,742 (52.4)	4,679 (53.7)	1,063 (47.6)	
Non-Hispanic Black	2,027 (18.5)	1,567 (18.0)	460 (20.6)	
Mexican American	2,348 (21.4)	1,839 (21.1)	509 (22.8)	
Other	834 (7.6)	634 (7.3)	200 (9.0)	
Education level, *n* (%)				<0.001
Less than high school	3,325 (30.4)	2,570 (29.5)	755 (33.8)	
High school or equivalent	2,592 (23.7)	2,043 (23.4)	549 (24.6)	
Above high school	5,034 (46.0)	4,106 (47.1)	928 (41.6)	
Marital status, *n* (%)				<0.001
Married	6,296 (57.5)	5,119 (58.7)	1,177 (52.7)	
Other	4,655 (42.5)	3,600 (41.3)	1,055 (47.3)	
BMI, Mean ± SD, kg/m^2^	28.42 ± 6.26	28.26 ± 6.05	29.05 ± 7.01	< 0.001
Physical activity, *n* (%)	6,257 (57.1)	5,059 (58.0)	1,198 (53.7)	0.001
Smoking status, *n* (%)	5,366 (49.0)	4,292 (49.2)	1,074 (48.1)	0.380
Alcohol consumption, *n* (%)				<0.001
Never	3,466 (31.7)	2,642 (30.3)	824 (36.9)	
Former	1,263 (11.5)	1,017 (11.7)	246 (11.0)	
Light	2,904 (26.5)	2,441 (28.0)	463 (20.7)	
Moderate	1,370 (12.5)	1,096 (12.6)	274 (12.3)	
Heavy	1,948 (17.8)	1,523 (17.5)	425 (19.0)	
Diabetes, *n* (%)	1,075 (9.8)	870 (10.0)	205 (9.2)	0.187
Hypertension, *n* (%)	3,511 (32.1)	2,811 (32.2)	700 (31.4)	0.353
DI-GM score, Mean ± SD	4.37 ± 1.45	4.42 ± 1.45	4.17 ± 1.41	<0.001
BGMS	2.03 ± 1.17	2.08 ± 1.18	1.86 ± 1.14	<0.001
UGMS	2.33 ± 1.00	2.34 ± 1.00	2.31 ± 1.02	0.329
Vitamin B1, Mean ± SD, mg	1.60 ± 0.88	1.61 ± 0.86	1.52 ± 0.95	<0.001
Vitamin B2, Mean ± SD, mg	2.08 ± 1.17	2.10 ± 1.16	1.99 ± 1.20	<0.001
Vitamin B6, Mean ± SD, mg	1.85 ± 1.12	1.88 ± 1.11	1.76 ± 1.17	<0.001
Vitamin B12, Mean ± SD, mg	5.28 ± 9.82	5.37 ± 10.47	4.91 ± 6.75	<0.001
Zinc, Mean ± SD, mg	11.61 ± 8.38	11.69 ± 8.10	11.30 ± 9.39	<0.001
Copper, Mean ± SD, mg	1.28 ± 1.27	1.29 ± 1.35	1.22 ± 0.88	<0.001

The DI-GM score comprises BGMS and UGMS. Abbreviations: BGMS, beneficial to gut microbiota score; BMI, body mass index; DI-GM, dietary index for gut microbiota; NHANES, National Health and Nutrition Examination Survey; SD, standard deviation; UGMS, unfavorable to gut microbiota score.

### Association between DI-GM and severe headache or migraine

As shown in Table 2, in the unadjusted model (Model 1), a higher DI-GM score was significantly associated with a lower prevalence of severe headache or migraine (OR: 0.89, 95% CI: 0.86–0.92; *p* < 0.001). After adjusting for age, sex, race/ethnicity, education level, marital status, and BMI (Model 2), this association remained significant (OR: 0.95, 95% CI: 0.91–0.98; *p* = 0.004). This relationship persisted after further adjustments for physical activity, smoking status, alcohol consumption, diabetes, and hypertension in Model 3 (OR: 0.95, 95% CI: 0.91–0.99; *p* = 0.010). In the fully adjusted model (Model 4), accounting for nutritional factors like vitamins B1, B2, B6, B12, zinc, and copper, higher DI-GM scores were still significantly associated with a reduced prevalence of severe headache or migraine, with each 1-point increase in DI-GM corresponding to a 5% reduction in prevalence (OR: 0.95, 95% CI: 0.91–0.99; *p* = 0.011). Furthermore, BGMS was significantly and inversely associated with prevalence of severe headache or migraine across all models (Model 1: OR = 0.85, 95% CI = 0.81–0.90, *p* < 0.001; Model 2: OR = 0.91, 95% CI = 0.86–0.95, *p* < 0.001; Model 3: OR = 0.91, 95% CI = 0.86–0.96, *p* = 0.002; Model 4: OR = 0.90, 95% CI = 0.85–0.96, *p* = 0.003). However, no association was observed between UGMS and prevalence of severe headache or migraine (all *p* > 0.05).

**Table 2:** Association between DI-GM and severe headache or migraine in NHANES 1999–2004.

Characteristic	Model 1			Model 2			Model 3			Model 4		
	OR	95% CI	*P-*value	OR	95% CI	*P-*value	OR	95% CI	*P-*value	OR	95% CI	*P-*value
DI-GM score	0.89	0.86, 0.92	<0.001	0.95	0.91, 0.98	0.004	0.95	0.91, 0.99	0.010	0.95	0.91, 0.99	0.011
BGMS	0.85	0.81, 0.90	<0.001	0.91	0.86, 0.95	<0.001	0.91	0.86, 0.96	0.002	0.90	0.85, 0.96	0.003
UGMS	0.97	0.93, 1.01	0.134	1.01	0.97, 1.06	0.467	1.02	0.97, 1.06	0.446	1.01	0.97, 1.06	0.578

Model 1: unadjusted.

Model 2: adjusted for age, sex, race/ethnicity, education level, marital status, and BMI.

Model 3: further adjusted for physical activity, smoking status, alcohol consumption, diabetes, and hypertension.

Model 4: additionally adjusted for nutrient intake (vitamins B1, B2, B6, B12, zinc, and copper).

The DI-GM score comprises BGMS and UGMS. Abbreviations: BGMS, beneficial to gut microbiota score; BMI, body mass index; CI, confidence interval; DI-GM, dietary index for gut microbiota; NHANES, National Health and Nutrition Examination Survey; OR, odds ratio; UGMS, unfavorable to gut microbiota score.

**Figure 2:**
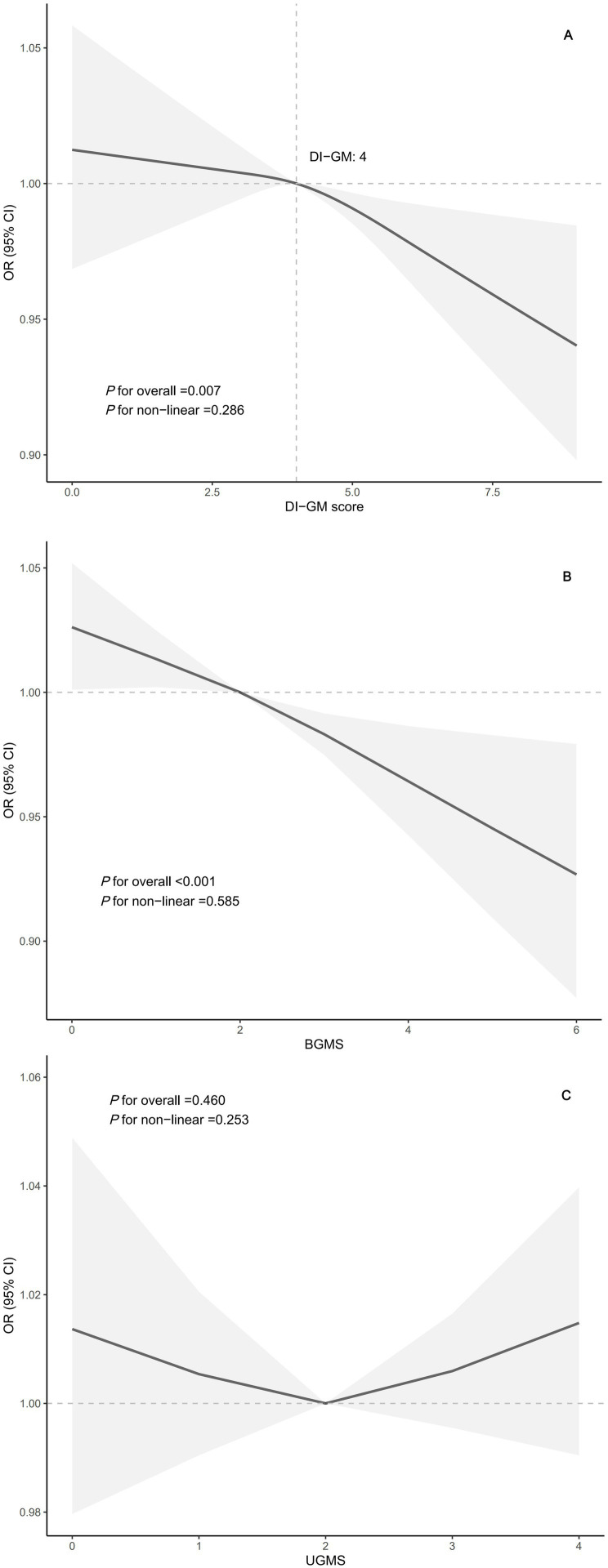
Association between DI-GM and severe headache or migraine in NHANES 1999–2004 by RCS. **(A)** Linear association between DI-GM score and severe headache or migraine. **(B)** Linear association between BGMS and severe headache or migraine. **(C)** Non-significant linear trend between UGMS and severe headache or migraine. The Model was adjusted for age, sex, race/ethnicity, education level, marital status, BMI, physical activity, smoking status, alcohol consumption, diabetes, hypertension, and nutrient intake (vitamins B1, B2, B6, B12, zinc, and copper). The DI-GM score comprises BGMS and UGMS. Abbreviations: BGMS, beneficial to gut microbiota score; BMI, body mass index; CI, confidence interval; DI-GM, dietary index for gut microbiota; NHANES, National Health and Nutrition Examination Survey; OR, odds ratio; RCS, restricted cubic spline; UGMS, unfavorable to gut microbiota score.

As shown in Figure 2, RCS revealed a linear relationship between DI-GM, BGMS and severe headache or migraine (DI-GM: *P* for non-linear = 0.286; BGMS: *P* for non-linear = 0.585), with a threshold effect identified at a DI-GM score of 4.

As shown in Table 3, in two-piecewise regression models, a significant inverse association was observed between DI-GM score and prevalence of severe headache or migraine in participants with DI-GM score ≥ 4 across all models (Model 1: OR = 0.85, 95% CI = 0.79–0.90, *p* < 0.001; Model 2: OR = 0.90, 95% CI = 0.84–0.96, *p* = 0.002; Model 3: OR = 0.91, 95% CI = 0.85–0.97, *p* = 0.005; Model 4: OR = 0.90, 95% CI = 0.85–0.97, *p* = 0.005), whereas no association was observed in those with DI-GM score < 4 (all *p* > 0.05).

**Table 3:** Association between DI-GM and severe headache or migraine using two-piecewise regression models in NHANES 1999–2004.

Model	DI-GM < 4			DI-GM ≥ 4		
	OR	95% CI	*P*-value	OR	95% CI	*P*-value
Model 1	0.91	0.78, 1.07	0.242	0.85	0.79, 0.90	< 0.001
Model 2	0.98	0.83, 1.16	0.843	0.90	0.84, 0.96	0.002
Model 3	0.98	0.83, 1.17	0.847	0.91	0.85, 0.97	0.005
Model 4	0.99	0.83, 1.18	0.872	0.90	0.85, 0.97	0.005

Model 1: unadjusted.

Model 2: adjusted for age, sex, race/ethnicity, education level, marital status, and BMI.

Model 3: further adjusted for physical activity, smoking status, alcohol consumption, diabetes, and hypertension.

Model 4: additionally adjusted for nutrient intake (vitamins B1, B2, B6, B12, zinc, and copper).

Abbreviations: BMI, body mass index; CI, confidence interval; DI-GM, dietary index for gut microbiota; NHANES, National Health and Nutrition Examination Survey;OR, odds ratio.

### Subgroup analyses

**Figure 3:**
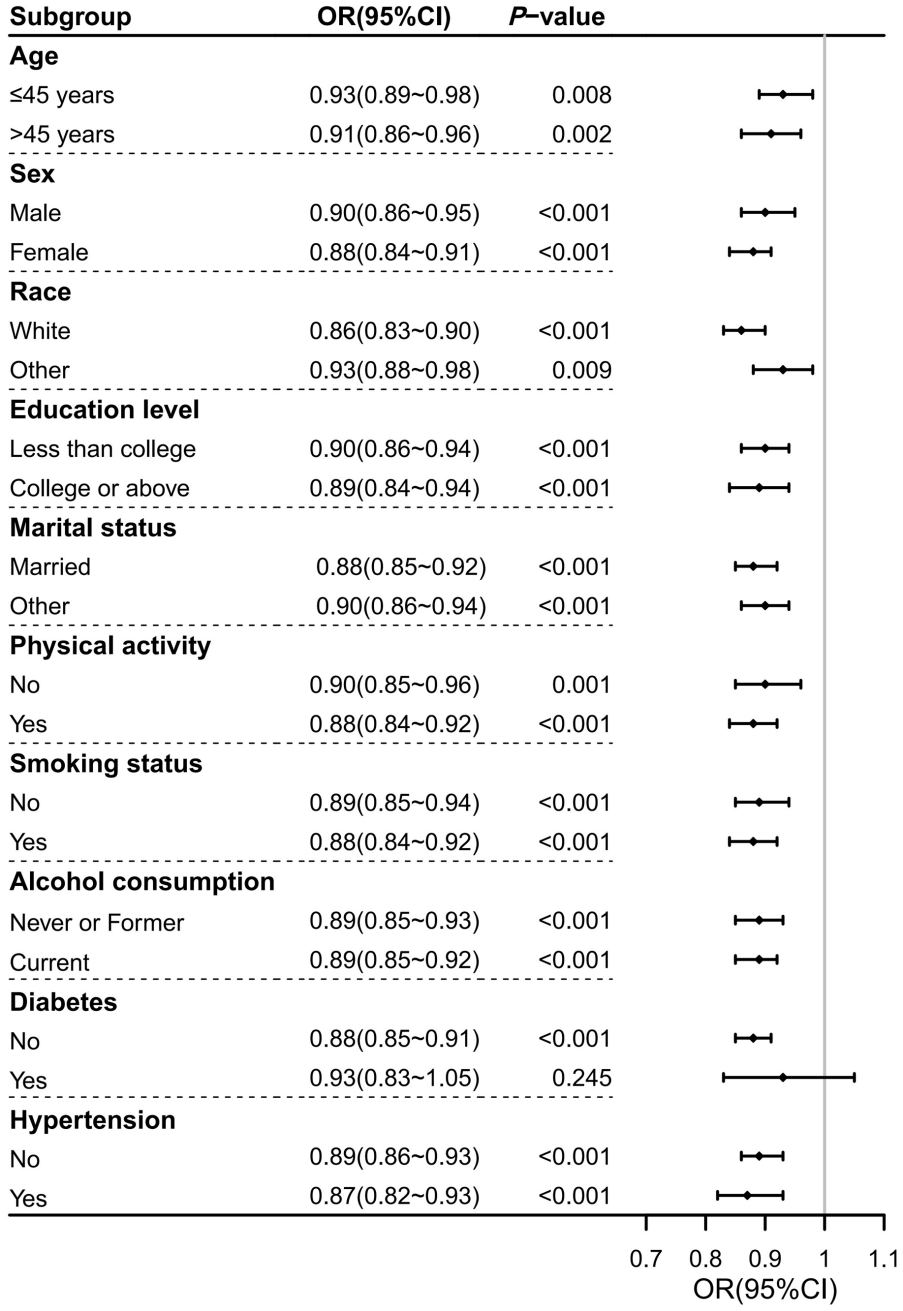
Forest plot of subgroup analyses on the association between DI-GM and severe headache or migraine in NHANES 1999–2004. Significant associations were observed across all subgroups except for the diabetes subgroup. Abbreviations: CI, confidence interval; DI-GM, dietary index for gut microbiota; NHANES, National Health and Nutrition Examination Survey; OR, odds ratio.

As shown in Figure 3, the association between DI-GM and prevalence of severe headache or migraine was not significant in the diabetes subgroup (OR = 0.93, 95%CI: 0.83–1.05; *p* = 0.245). However, in all other subgroups, this association remained significant (*p* < 0.01). OR values across these subgroups showed minimal variation, with the lowest OR observed in the non-Hispanic White subgroup (OR = 0.86) and the highest in the age ≤ 45 years and other race subgroups (OR = 0.93), confirming a stable and reliable association between DI-GM and prevalence of severe headache or migraine.

## Discussion

This study first demonstrated a significant inverse association between DI-GM and prevalence of self-reported severe headache or migraine. Participants in the headache group had lower DI-GM, with higher scores linked to reduced prevalence of severe headache or migraine. Multivariable logistic regression analyses confirmed this relationship, showing that each 1-point increase in the DI-GM was associated with a 5% reduction in the prevalence of severe headache or migraine, even after adjusting for multiple demographic, lifestyle, and nutritional factors. A dose–response relationship was observed, with a threshold at DI-GM score of 4, above which the prevalence of severe headache or migraine decreased significantly. Subgroup analyses confirmed the consistency of this association across different examined subgroups, underscoring the robustness of the findings.

The role of diet in shaping gut microbiota and its impact on migraine has been extensively studied. Fermented dairy products, identified as beneficial to gut microbiota within DI-GM, have been shown to significantly enhance microbiota diversity and reduce inflammatory markers (8). Arzani et al. (29) further highlighted the importance of the gut-brain axis (GBA) in the pathophysiology of migraine, particularly through immune regulation, inflammation, and neural communication. In contrast, refined grains, classified as detrimental to gut microbiota, are a prominent feature of the Western diet. Overconsumption of refined grains can lead to hyperglycemia, promoting gut- and neuro-inflammation, both of which are linked to migraine (30). Similarly, diets with a high fat-to-carbohydrate ratio reduce gut microbiota diversity (29), exacerbating inflammation and potentially increasing migraine risk. Dietary interventions, such as increasing fiber and probiotics, offer promising strategies to reduce migraine frequency and severity by targeting the GBA (5, 31). Further research should focus on understanding the microbial and immune mechanisms underlying migraine, ultimately enabling more personalized and effective interventions.

The DI-GM quantifies diets that are either beneficial or unfavorable to gut microbiota, with changes in DI-GM subsequently affecting gut microbiota diversity. It is generally believed that alterations in gut microbiota, or gut dysbiosis, play a key role in neurological diseases, including migraine, through the microbiota-gut-brain axis, which mediates bidirectional communication between the central nervous system (CNS) and the gastrointestinal (GI) tract (28, 31, 32). Migraine development seems to be partially related to gut dysbiosis, which can lead to a reduction in SCFAs production and to a concomitant increase in gut-derived inflammatory cytokines, thereby influencing CNS activities (33). Pro-inflammatory cytokines, such as interleukin (IL)-1β, IL-6, and tumor necrosis factor (TNF)-α, rise during migraine attacks, sensitizing pain pathways and increasing gut permeability, worsening neuroinflammation (4, 5). Microbial metabolites, including SCFAs, could mitigate inflammation and reduce migraine frequency (4). Therefore, targeting GBA through diet may provide novel avenues for migraine management.

Our findings that higher DI-GM scores correlated with reduced prevalence of self-reported severe headache or migraine align with growing evidence linking diet, inflammation, and migraine (34). Liu et al. (35) demonstrated that lower dietary inflammatory potential is associated with reduced migraine incidence, especially among adult women. Studies on dietary inflammatory indexes (DII) further underscore that inflammation-triggering foods, such as refined grains and high-fat items in Western diets, exacerbate systemic inflammation and may increase migraine susceptibility (36). Additionally, the Alternative Healthy Eating Index (AHEI), which emphasizes anti-inflammatory and antioxidant-rich foods like fruits, vegetables, whole grains, and nuts, has shown potential benefits in reducing migraine and other inflammation-related conditions by prioritizing healthy dietary components over pro-inflammatory ones (37). The Food Inflammation Index (FII), as outlined by Wang et al. (36), identifies key inflammatory components like saturated fats, often linked to migraine. Findings from Bakhshimoghaddam et al. (38) on the Dietary and Lifestyle Inflammation Score (DLIS) show a significant association between high inflammation potential diets and chronic migraine prevalence, reinforcing the role of dietary inflammation in migraine risk. Interestingly, our study found no direct link between alcohol consumption and severe headache or migraine, though DI-GM was significantly associated with headache across all alcohol consumption categories. These findings align with a large meta-analysis showing no negative relationship between alcohol and primary headaches, challenging the assumption that alcohol consumption exacerbates them (39). In addition, the absence of a significant association in the diabetes subgroup may be attributed to two factors: as Ha et al. (40) reported, diabetes is negatively associated with migraine occurrence, while migraine correlates positively with diabetes, and the influence of dietary interventions on blood glucose control and gut microbiota modulation. Therefore, our study positions the DI-GM as a powerful tool for linking diet and headaches through gut microbiota health, providing targeted nutritional strategies to modulate microbiota as a novel approach for managing severe headache or migraine.

Our study has several limitations. First, the cross-sectional design prevents establishing causality between DI-GM and severe headache or migraine. Longitudinal or prospective studies are needed to confirm the temporal associations. Second, severe headache or migraine was determined based on self-reported answers to the questionnaire, and the headache classification was not independently verified by medical personnel. Third, a single 24-h recall may not capture long-term patterns but reflects typical adult diets, which are generally stable unless influenced by health or lifestyle changes. Fourth, this study lacked direct gut microbiota data, unavailable in NHANES and unfeasible in large population-based surveys due to invasive sampling and sequencing. However, DI-GM is a practical, diet-based surrogate, ideal for large epidemiological datasets like NHANES. Fifth, reliance on self-reported survey data may introduce recall and social desirability biases, and despite adjusting for multiple confounders, residual confounding from unmeasured factors may still exist. Lastly, the study did not take into account the use of medications to treat headaches, especially those that affect gut microbiota. However, since DI-GM is a diet-based index, it is unlikely to be significantly influenced by medication use, thus minimizing its potential to confound the results.

## Conclusion

The DI-GM was negatively associated with the prevalence of self-reported severe headache or migraine in U.S. adults, particularly when scores exceeded 4. These findings suggest that dietary interventions targeting gut microbiota could be a promising strategy for managing and preventing migraine episodes. Further longitudinal studies are needed to validate these results and explore causal mechanisms.

## Data Availability

The original contributions presented in the study are included in the article/supplementary material, further inquiries can be directed to the corresponding author.
